# Ultrasound-Guided Nerve Block with Botulinum Toxin Type A for Intractable Neuropathic Pain

**DOI:** 10.3390/toxins8010018

**Published:** 2016-01-08

**Authors:** Young Eun Moon, Jung Hyun Choi, Hue Jung Park, Ji Hye Park, Ji Hyun Kim

**Affiliations:** Department of Anesthesiology and Pain Medicine, College of Medicine, The Catholic University of Korea, Seoul St. Mary’s Hospital, 222 Banpo-Daero, Seoul 137-701, Korea; momo0910@catholic.ac.kr (Y.E.M.); js031355@hanmail.net (J.H.C.); jh0192@hanmail.net (J.H.P.); delita605@hotmail.com (J.H.K.)

**Keywords:** botulinum toxin type A, diabetic polyneuropathy, nerve block, postherpetic neuralgia

## Abstract

Neuropathic pain includes postherpetic neuralgia (PHN), painful diabetic neuropathy (PDN), and trigeminal neuralgia, and so on. Although various drugs have been tried to treat neuropathic pain, the effectiveness of the drugs sometimes may be limited for chronic intractable neuropathic pain, especially when they cannot be used at an adequate dose, due to undesirable severe side effects and the underlying disease itself. Botulinum toxin type A (BoNT-A) has been known for its analgesic effect in various pain conditions. Nevertheless, there are no data of nerve block in PHN and PDN. Here, we report two patients successfully treated with ultrasound-guided peripheral nerve block using BoNT-A for intractable PHN and PDN. One patient had PHN on the left upper extremity and the other patient had PDN on a lower extremity. Due to side effects of drugs, escalation of the drug dose could not be made. We injected 50 Botox units (BOTOX^®^, Allergan Inc., Irvine, CA, USA) into brachial plexus and lumbar plexus, respectively, under ultrasound. Their pain was significantly decreased for about 4–5 months. Ultrasound-guided nerve block with BoNT-A may be an effective analgesic modality in a chronic intractable neuropathic pain especially when conventional treatment failed to achieve adequate pain relief.

## 1. Introduction

Neuropathic pain has been defined as “pain initiated or caused by a primary lesion or dysfunction in the nervous system” by the International Association for the Study of Pain [1]. There are various neuropathic pain conditions, which include postherpetic neuralgia (PHN), painful diabetic neuropathy (PDN), trigeminal neuralgia, and so on. Since neuropathic pain is correlated with complex mechanisms, various drugs have been tried in order to treat neuropathic pain, such as anticonvulsants, antidepressants, opioids, topical agents, *etc*. [2]. However, the effectiveness of the drugs sometimes may be limited for chronic intractable neuropathic pain, especially when they cannot be used at an adequate dose, due to undesirable side effects and the underlying disease itself [3].

Botulinum toxin type A (BoNT-A) has been used for conditions like hemifacial spasm, cervical dystonia, and blepharospasm associated with excessive muscle tension. Recently, BoNT-A is also known for an analgesic effect in various pain conditions [4]. Nevertheless, there are no data of nerve block in PHN and PDN.

Here, we report two patients successfully treated with ultrasound-guided nerve block using BoNT-A for intractable PHN and PDN.

## 2. Case Reports

### 2.1. Case 1

**Figure 1:**
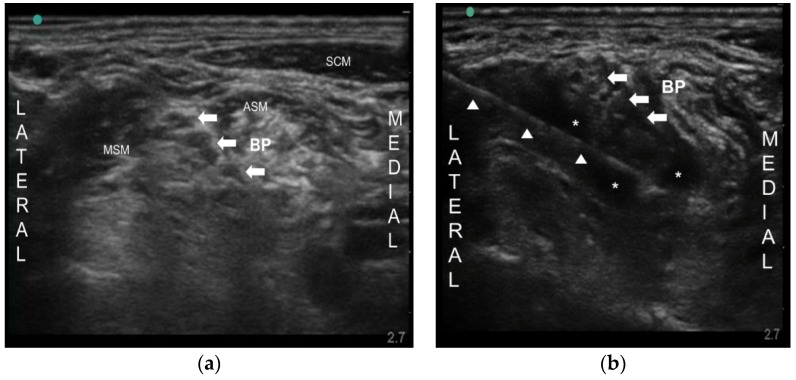
(**a**) Ultrasound image of the brachial plexus between the anterior and middle scalene muscle. Arrows indicate brachial plexus (BP); (**b**) Ultrasound image of spreading the drugs around the brachial plexus. Arrowheads indicate the needle. Arrows indicate BP. ASM anterior scalene muscle, MSM middle scalene muscle, SCM sternocleidomastoid muscle, * injected drug surrounding the BP.

A 63-year-old woman was referred to our pain clinic with PHN for one year. She was an acute myelogenous leukemia patient and had undergone allogeneic peripheral blood stem cell transplantation in the preceding year. She was a terminal cancer patient. Three months after herpes zoster from left shoulder to all fingers, she was diagnosed as PHN by dermatologist. She complained of a very sharp and burning pain on a whole left arm (from shoulder to all fingers, C5–8 dermatome). Hyperalgesia, tactile allodynia, and paresthesia were accompanied. Her visual analog scale (VAS) was 9/10. She was prescribed tacrolimus 0.5 mg/day for immunosuppression, neurontin 600 mg/day, nortriptyline 10 mg/day, and fentanyl patch 12 μg for PHN. Nonetheless, her pain was not controlled by medication (VAS 8/10). Loading neurontin 900 mg/day was attempted, but failed due to severe nausea and vomiting. She was consulted by a gastroenterologist because of poor oral intake with a cachexic condition and gastrointestinal distress. An ultrasound-guided brachial plexus block (BPB) was attempted. After sterilization, a high frequency linear transducer was positioned in the transverse plane to identify the scalene muscles and the brachial plexus that is sandwiched between the anterior and middle scalene muscles. The needle (BD, Singapore, Singapore, 50 mm 25G) was inserted in-plane into the interscalene groove between the anterior and middle scalene muscles using 10 mL of 0.4% lidocaine (Figure 1). The VAS decreased from 8/10 to 3–4/10 for only 3 days. Then, cervical epidural block (C6–7 level, left side) with 4 mL of 0.4% lidocaine and 1 mg of dexamethasone was performed under fluoroscopy. Her pain was relieved (VAS from 8/10 to 2–3/10) just for a week. Therefore, after obtaining patient’s consent form and institutional review board approval (KC13ZISE0739), we injected 10 mL of 0.1% bupivacaine with 50 Botox units (BOTOX^®^, Allergan Inc., Irvine, CA, USA) around brachial plexus. Since 50 Botox units were reconstituted in 2 mL of 0.9% normal saline, total volume injected was 12 mL (Figure 1). Subsequently, the VAS was reduced from 8/10 to 2–3/10, and at the five-month follow-up, her pain was well controlled (VAS 3/10) and she was satisfied with BoNT-A treatment.

### 2.2. Case 2

**Figure 2:**
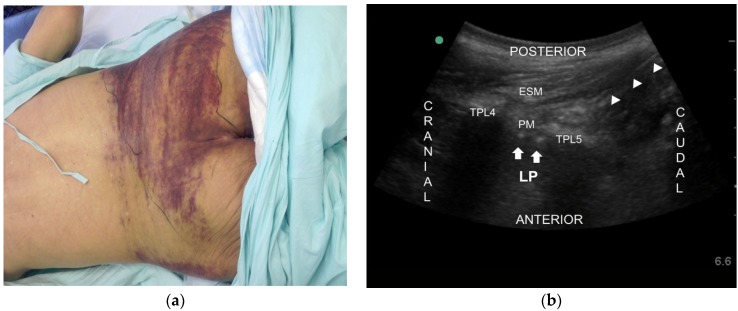
(**a**) Picture of severe bruise after fall due to dizziness of adverse effect of medication in Case 2; (**b**) Ultrasound image of the lumbar plexus block in the posterior part of the psoas muscle (PM). Arrows indicate lumbar plexus (LP). Arrowheads indicate the needle. ESM erector spinae mescle, PM psoas muscle, TPL4 transverse process of L4, TPL5 transverse process of L5.

A 74-year-old man visited our pain clinic for control of a five-year history of left diabetic leg pain. He had suffered from diabetes over 25 years and undergone a right below knee amputation due to a diabetic foot two years earlier. Several comorbidities were present and he had undergone bypass surgery on both legs due to atherosclerosis obliterans eight years earlier. Furthermore, he had suffered a cerebrovascular attack six years prior to his clinic visit, which left sequelae of hemiparesis on his right side. The patient had persistent and severe pain with a burning and tingling sensation from left knee to all toes (VAS 10/10). Pregabalin 225 mg/day, acetaminophen plus tramadol 500 plus 60 mg/day, and nortriptyline 10 mg/day were prescribed. However, his pain was still severe (VAS 9/10). We tried to increase dosage of drug (Pregabalin 300 mg/day) and add opioids (oxycodon 10 mg/day) but in vain. A few days later, he revisited complaining of a fall due to dizziness (Figure 2a). We could not caudal epidural block because of a severe bruise. So, we did an ultrasound-guided lumbar plexus block (LPB) with 10 mL of 0.4% lidocaine. With the patient in the prone position, a low frequency curved transducer was positioned approximately 3–4 cm lateral and parallel to the lumbar spine (over the L4 transverse process). The psoas muscle was seen between the transverse processes and part of the lumbar plexus was also seen as hyperechoeic dots in the posterior part of the psoas muscle. The needle (BD, Singapore, Singapore, 60 mm 24G) was inserted in-plane from the caudal end and advanced into the posterior part of the psoas muscle. After negative aspiration through the needle, the drug was injected in real time (Figure 2b). The VAS decreased from 9/10 to 4/10 for only seven days. After obtaining the patient’s consent form and institutional review board approval (KC13ZISE0739), an ultrasound-guided LPB was performed again with 0.1% bupivacaine 10 mL and 50 Botox units (BOTOX^®^, Allergan Inc., Irvine, CA, USA). Since 50 Botox units were reconstituted in 2 mL of 0.9% normal saline, total volume injected was 12 mL (Figure 2b). The VAS of 9/10 subsequently decreased to 2/10. At four months follow-up observation, the patient’s pain was well controlled (VAS 2/10).

## 3. Discussion

In these cases, BoNT-A for BPB and LPB alleviated pain effectively on intractable PHN and PDN patients especially who showed side effects and were reluctant to conventional treatment. Diagnostic nerve block with local anesthetics only decreased pain for a short time in two neuropathic patients. After nerve plexus block with BoNT-A, pain relief was sustained for about 4–5 months.

For the treatment of PHN and PDN, antidepressants, anticonvulsants, topical patch, opioids, and new medical treatments are recently introduced, but those are still unsatisfactory for pain control and quality of life [5,6].

Although there have been many studies about BoNT-A’s analgesic effects in various neuropathic pain syndromes which include PHN, trigeminal neuralgia, post-traumatic neuralgia, carpal tunnel syndrome, PDN, complex regional pain syndrome, phantom limb and stump pain, and occipital neuralgia, there have been only two studies that showed application of BoNT-A into nerve block for occipital neuralgia (ON) among them [7]. According to Kapural *et al.* [8], in six patients of ON who were treated conservatively, BoNT-A group (50 Botox units (BOTOX^®^, Allergan Inc., Irvine, CA, USA) for each block; 100 Botox units if bilateral) for occipital nerve block (ONB) experienced significantly decreased pain and improved daily activities compared to the bupivacaine-treated group. However, only two of six patients received diagnostic greater ONB using electrical stimulation in this retrospective case series. The other pilot study revealed that BoNT-A (50 Botox units (BOTOX^®^, Allergan Inc., Irvine, CA, USA)) ONB in six ON patients improved sharp and/or shooting pain and quality of life for 12 weeks, prospectively [9]. However, authors performed greater and lesser occipital nerve blocks with a blind technique in this study. Unlike the previous two studies, we injected 50 Botox units (BOTOX^®^, Allergan Inc., Irvine, CA, USA) into brachial plexus and lumbar plexus using ultrasound in PHN and PDN. The use of the ultrasound-guided nerve block can offer better accuracy and fewer complications than blind procedures because of the ability to view nerves and the surrounding soft tissue structures, such as muscles and vessels without any radiation exposure. Furthermore, the position of the needle tip and the spread of the drug injection can be observed in real-time. Therefore, the use of ultrasound with a BoNT-A nerve block can prevent unnecessary muscle relaxation around the target nerve.

Until now, there has not been any study that has demonstrated the use of BoNT-A for nerve blocks in PHN and PDN. There were five studies for PHN and one study for PDN using botulinum toxin. Those all used methods of intradermal or subcutaneous injections over multiple sites (40 injections for the maximum) [7,10,11,12]. Thus, the main adverse effect of those studies was severe pain caused by intradermal or subcutaneous injections. Moreover, some patients dropped out of the study due to intolerable pain by multiple injections [12]. In our cases, nerve blocks using BoNT-A did not require additional pain control prior to injection.

The analgesic mechanism of BoNT-A may be due to inhibition of secretion of the pain control neurotransmitter, calcitonin gene-related peptide, glutamate and substance P in the primary afferent nerve fibers [13,14]. Additionally, BoNT-A may reduce activity of TRPV1, which is involved in integrating noxious stimuli [15]. However, the mechanism of action of BoNT is, as yet, inconclusive.

In conclusion, an ultrasound-guided nerve block with BoNT-A may be a useful and alternative treatment modality for chronic intractable neuropathic pain conditions involving PHN and PDN, especially those that do not respond well to conventional therapy. More studies including long-term follow-up, double-blind investigations, and a comparison of different dosage and injection techniques are needed in the future.
